# Exosomal miRNAs as novel avenues for breast cancer treatment

**DOI:** 10.3389/fgene.2023.1134779

**Published:** 2023-03-22

**Authors:** Tejveer Singh, Mahesh Kaushik, Lokesh Chandra Mishra, Chesta Behl, Vijay Singh, Hardeep Singh Tuli

**Affiliations:** ^1^ Translational Oncology Laboratory, Department of Zoology, Hansraj College, Delhi University, New Delhi, India; ^2^ Radiation and Cancer Therapeutics Lab, School of Life Sciences, Jawaharlal Nehru University, New Delhi, India; ^3^ Immunology and Infectious Disease Biology Lab, CSIR-Institute of Genomics and Integrative Biology, New Delhi, India; ^4^ Department of Biotechnology, Maharishi Markandeshwar Engineering College, Maharishi Markandeshwar (Deemed to be University), Ambala, India

**Keywords:** breast cancer, exosome, miRNA, diagnosis, metastasis

## Abstract

Breast cancer is the most commonly diagnosed cancer and a leading cause of death in women worldwide. It is a heterogeneous disease, as shown by the gene expression profiles of breast cancer samples. It begins in milk-producing ducts, with a high degree of diversity between and within tumors, as well as among cancer-bearing individuals. The enhanced prevalence of breast cancer is influenced by various hormonal, lifestyle, and environmental factors, and very early onset of the disease correlates strongly with the risk of local and distant recurrence. Many subtypes are difficult to treat with conventional therapeutic modalities, and therefore, optimal management and early diagnosis are the first steps to minimizing the mortality linked with breast cancer. The use of newer methods of nanotechnology extends beyond the concept of synthesizing drug delivery mechanisms into the creation of new therapeutics, such as delivering chemotherapeutics with nanomaterial properties. Exosomes, a class of nanovesicles, are emerging as novel tools for deciphering the patient-specific proteins and biomarkers across different disease models, including breast cancer. In this review, we address the role of exosomal miRNA in breast cancer diagnosis and treatment.

## GRAPHICAl ABSTRACT

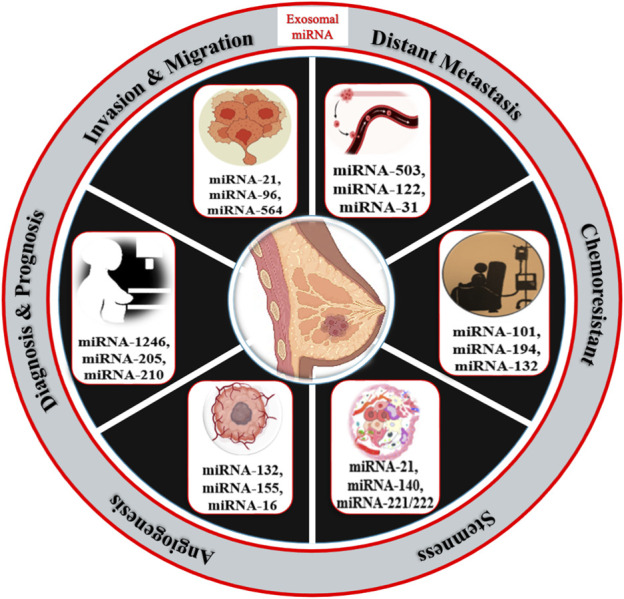

## Highlights

➣ Exosomal miRNA role in the progression and diagnosis of breast cancer
➣ Exosomal miRNA role in chemoresistance of breast cancer
➣ Exosomal miRNA role in the different hallmarks of breast cancer

## Introduction

New estimates suggest that about one in 300 women are diagnosed with an aggressive form of breast cancer before age 40 (DeSantis et al., 2019). Breast cancer is classified as either specific [20%–25%] or non-specific ductal carcinoma [60%–75%] subtypes, which include papillary, mucinous, lobular, and tubular tumors.

Its molecular classification is on the basis of the presence and absence of human epidermal growth factor 2 [HER2], estrogen or progesterone receptor [ERBB2] (hormone receptor negative/ERBB2− [70%], positive/ERBB2+ [15%–20%], and triple negative in which all the three markers are absent [15%]), basal-like [ER-/HER2−], HER2 enriched [HER2+], and combined luminal A and B [ER+/HER2−] (Johnson et al., 2020).

The triple-negative breast cancer (TNBC) subtype has a poor prognosis and a maximum rate of systemic recurrence. Various approaches have been applied to eradicate it, including modulation of the tumor microenvironment to increase CTL activity and other immunotherapy-based approaches like immune checkpoint inhibition by neutralizing antibodies and neoadjuvant-based immunotherapy (Jia et al., 2017).

These locally advanced and metastatic lesions show extensive nodal involvement and an inflammatory phenotype. The prognosis of these lesions is often unfavorable; despite an aggressive treatment regime, it eventually leads to an enhanced mortality rate (Hapach et al., 2021).

Newer innovations in systemic therapy, such as surgical procedures, radiotherapy, and the development of new advanced targeted agents, have improved the clinical outcomes of this malignancy. Therefore, optimal management and early diagnosis are the first key steps toward minimizing the mortality linked with this malignancy. Targeting molecular pathways and elucidating the molecular cascade and their relationship with other signaling molecules has led to the evolution of practical combination therapies that have been proven successful to a larger extent.

Currently, available cancer therapies are limited to surgery, radiation, and chemotherapy, with a high risk of bystander effect and damage to normal tissues. These conventional therapies have side effects and chemo/radioresistant and other toxicity-related issues. Nanotechnology-based methods selectively target cancerous cells, minimizing therapy side effects and enhancing the probability of survival. Exosomes, a class of nanovesicles that mediate cellular communications *via* delivering many types of biomolecules [oncogenes, protein DNA, and RNA, including different pharmacological compounds], are being explored to model the patient-specific proteins and biomarkers across different disease models, including breast cancer. An interesting aspect of these nanocarriers is that they can serve as informative sources of novel biomarkers, and their cargo can deliver therapeutic molecules across the target sites. Many studies have signified the role of exosomes in breast cancer biology, including the identification of signature molecules and regulation of the breast cancer tumor microenvironment. This comprehensive review addresses the role of exosomal miRNA as a diagnostic tool and treatment for breast cancer therapy.

## The emerging role of liquid biopsy for breast cancer diagnosis

Identification of stage-specific cancer biomarkers is a prerequisite for early detection. Owing to the heterogeneous nature, small sample size, and varied genomic profiles of tumors, conventional biopsies often fail to reflect the whole nature of primary or secondary metastasis. Moreover, frequent tissue sampling from cancer patients for the identification of tumor-associated genetic changes, therapy responses, and investigating tumor dynamics is also a major concern. Therefore, there is an unmet need for novel low-cost and non-invasive sampling techniques and methods that could improve early detection and screening. In this context, identification of circulating tumor cells (CTCs) separated from the original tumor bulk has been considered a gold standard for identifying tumor-related evidence. However, due to the reduced availability of CTCs, isolation of genetic materials from the bloodstream or any other biological fluid is an alternate option that is equally convenient and minimally invasive.

Compared to direct tumor biopsies, collection from bioliquids seems an attractive alternative source for clinical application. In this context, the utility of exosomes has recently been reviewed in different tumor models. Exosome-based technologies offer several advantages over the existing traditional methods of biopsies as they show a universal presence across different biofluids, and the ease of isolation and characterization of their cargo makes them attractive tools (Wang et al., 2021).

**FIGURE 1:**
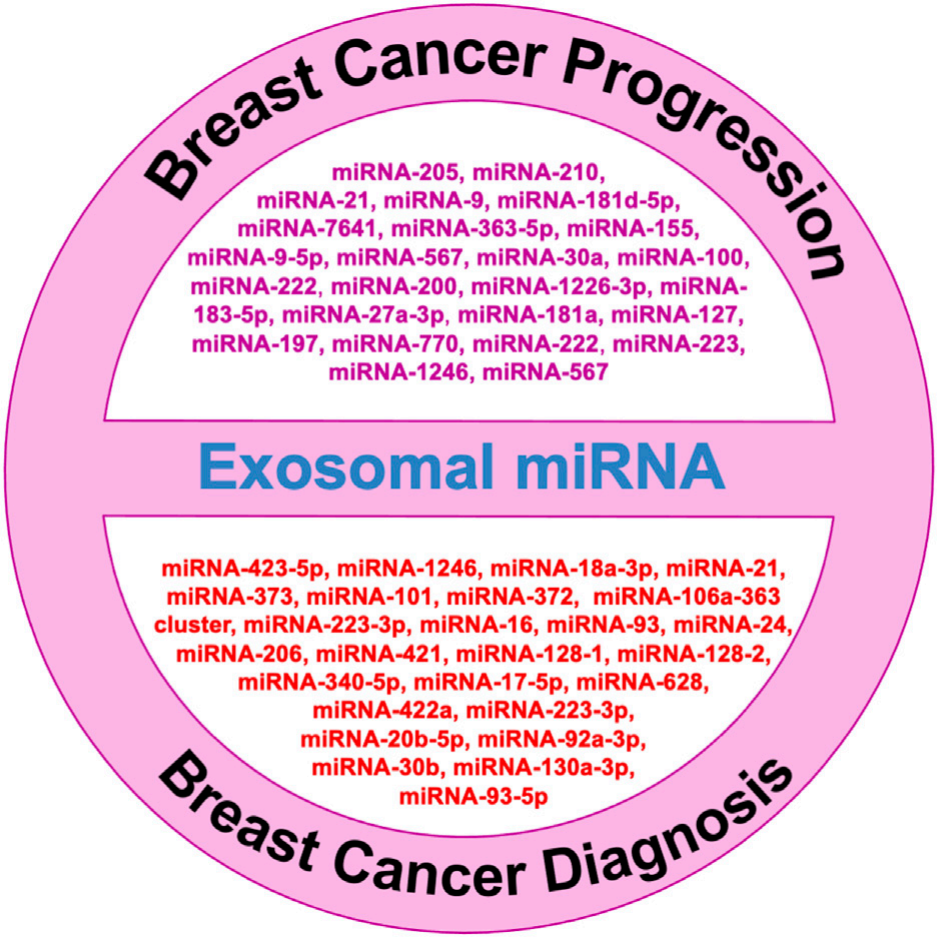
Role of exosomal microRNAs in breast cancer diagnosis and progression.

Breast cancer is one of the topmost threats to women’s health as diverse factors participate in tumorigenesis events. Among them, cell proliferation, stemness, metastasis, angiogenesis, epithelial-to-mesenchymal transition, and chemoresistance are major contributors to malignancy and reoccurrence. MicroRNAs that are specifically packed and secreted in exosomes are known as “exosomal microRNAs [miRNAs].” In contrast to different vesicular populations present in the biological fluids like apoptotic bodies and large vesicles, these exosomes more clearly represent the information present on the tumor. In this review, we summarize the utility and potential of small nanovesicles (exosomes) in different biological fluids, with a special focus on breast cancer (Figure 1), and show the different exosomal miRNAs in breast cancer progression.

## Exosomal microRNAs in BC diagnosis

Cancer patients have more tumor-derived exosomes in circulation than healthy individuals (Tavoosidana et al., 2011)**.** Exosomal miRNAs in blood have the potential to be new circulating biomarkers for early detection and diagnosis of many cancers, including breast cancer (Ogata-Kawata et al., 2014). Figure 1 shows some exosomal miRNAs in the diagnosis of breast cancer. Exosomal miR-1246 and miR-21 were reported in plasma as functional measures for breast cancer diagnosis (Hannafon et al., 2016; Singh et al., 2021). Li et al. (2018) reported an exosomal miR106a-363 cluster as a novel diagnostic biomarker in breast cancer (Li et al., 2018). Of them, four exosomal miRNAs are plasma-derived [miR-106a-5p, miR-92a-2-5p, miR-106a-3p, and miR-20b-5p], while four are serum-derived [miR-20b-5p, miR-106a-5p, miR-92a-2-5p, and miR-106a-3p] and showed higher expression in breast cancer patients compared with healthy controls (Stevic et al., 2018). The expression levels of exosomal miR-101 and miR-372 were higher in the serum of 50 breast cancer patients than in 12 healthy individuals (Eichelser et al., 2014). Of 435 breast cancer patients, 224 were TNBC patients, and 211 were HER-2 positive. Analysis of their plasma for exosomal miRNA revealed 13 miRNAs were lower and five were higher in HER-2 positive compared with the levels of TNBC patients (Stevic et al., 2018). The expression levels of miR-223-3p of invasive ductal carcinoma [IDC] patients were showing significantly many fold changes from ductal carcinoma in situ [DCIS] patients as well as from healthy controls (Yoshikawa et al., 2018). Ni et al. (2018) also reported when the expression of exosomal miR-30b, miR-16, and miR-93 were checked in 42 DCIS, 111 IDC patients, and 39 healthy individuals, out of them exosomal miR-16 show significant higher expression in plasma of BC patients as well as in DCIS patients than healthy control while exosomal miR-93 showed higher expression in DCIS patients as compared with IDC patients (Ni et al., 2018).

## Exosomal microRNAs in BC for chemoresistance

Almost 90% of chemotherapy failure occurs due to long-term and repetitive usage of similar types of drugs, leading to the chemoresistance that is a major hurdle for breast cancer treatment. There is a need to understand the molecular mechanism in chemoresistance to minimize the recurrence rate. Many studies showed the role of exosomal miRNA in the induction of chemoresistance in breast cancer (Tang et al., 2021). Here, we review some signaling pathways that are specifically targeted by exosomal miRNA to regulate the particular drug sensitivity of breast cancer (see Table 1).

**TABLE 1:** Role of exosomal miRNAs in drug resistance.

Exosomal miRNA	Drug	Molecular targets	Mechanism	Reference
miR-221-3p, miR-25, miR-505, miR-34a, miR-181a, and miR-126a	Doxorubicin	PI3K, Akt, ISL, HDAC1, HSP70, K246, Bax, Bcl-2, and IL-33/IL13	↓ Proliferation and autophagy, ↑ Apoptosis, and modify tumor microenvironment	Clarke et al. (2001), Yamamoto et al. (2011), Zhu et al. (2013), Wang et al. (2014), Wu et al. (2014), Deng et al. (2017), Hu et al. (2018), Chen et al. (2020), and Pan et al. (2020)
miR-194, miR-132, miR-24, and miR-302b	Cisplatin	MeCP2, FIH1, BimL, E2F1, and ATM	Chemoresistance, EMT and stemness, and ┬ cell life cycle	Pogribny et al. (2010), Bockhorn et al. (2013), Cataldo et al. (2016), and Hu et al. (2018)
miR-301, miR-101, miR-320a, miR-214, and miR-451a	Tamoxifen	PTEN, MAGI-2, Akt, ARPP-19, ERR5, cMyc, Cyclin D1, UCP2, and Erα/14-3-3C	Resensitize tumor, ↑ apoptosis, and ┬ autophagy	Sachdeva et al. (2011), Shi et al. (2011), Lü et al. (2015), and Liu et al. (2016)
miR-30c, miR-125, miR-125b, miR-200, and miR-16	Taxane	BAK1, Sema4C, ZEB1/2, E-cadherin, and BCL-2	↑ Apoptosis and ┬ Autophagy	Cochrane et al. (2009), Shi et al. (2011), Kastl et al. (2012), Zhang et al. (2014), Yang et al. (2015), and Cataldo et al. (2016)
miR-328	Mitoxantrone	BCRP and ABCG2	Modulates drug deposition	Pan et al. (2009)
miR-221/222 and miR-101	Fulvestrant	TGF-β, β-Catenin, and EZH2	Chemoresistance	Rao et al. (2011) and Sachdeva et al. (2011)
miR-16 and miR-205-5p	Trastuzumab	ERBB2, p63, EGFR, FUBP1, and Cyclin J	↑ Cytotoxic effect of the drug	De Cola et al. (2015) and Venturutti et al. (2016)
Let-7	Verapamil	Ras, ESR1, CASP3, and HMGA2	↑ Chemoresistance, and modulate receptor expression EMT progression	Yang et al. (2015) and Guo et al. (2019)

↑- Increase/upregulation; ↓- Decrease/Downregulation; ┴- Inhibit/Prevent.

### Topoisomerase interactive agents

Doxorubicin is one of the commonly used drugs that intercalate into the DNA double helix, inhibit topoisomerase II enzyme activity, and attack mitochondrial and genomic DNA *via* ROS induction (van der Zanden et al., 2021). A previous study revealed a correlation between doxorubicin resistance and miRNA: around 309 miRNAs decreased, and 66 miRNAs increased (Chen et al., 2018). Akt is mainly involved in the signaling transduction pathway to modulate cell proliferation and DNA repair. It suppresses apoptosis, improves cell survival, and plays a significant role in chemoresistance (Yamamoto et al., 2011). Exosomal miR-221-3p regulates the PI3K/Akt/phosphoinositide-3-kinase regulatory subunit 1 [PIK3R1] to acquire the doxorubicin resistance (Pan et al., 2020). Meanwhile, exosomal miR-505 restores doxorubicin sensitivity by suppressing Akt3 activity and increasing apoptosis (Yamamoto et al., 2011). Mitoxantrone is also a commonly used topoisomerase II inhibitor, a chemotherapeutic drug that blocks the cell cycle (Evison et al., 2016). Increased expression of exosomal miR-328 was linked with increased sensitization of MCF-7/MX100 cells to mitoxantrone *via* downregulating the breast cancer resistance protein (BCRP/ABCG2) (Pan et al., 2009).

### Platinum analogs: Cisplatin

Cisplatin causes double-strand DNA breaks by crosslinking *via* direct binding to DNA (Dickson et al., 2011)**.** In cisplatin-resistant breast cancer cell lines, exosomal miR-194 and exosomal miR-132 inhibit the methyl-CpG-binding protein 2 [MECP2] (Cataldo et al., 2016). Some exosomal miRNAs downregulate chemoresistance (Hu et al., 2018). After cisplatin treatment, exosomal miRNA-302b causes cell cycle arrest and induces apoptosis (Bockhorn et al., 2013).

### Antimicrotubule agents

Paclitaxel and docetaxel are tricyclic compounds under taxane that target the microtubules resulting in defects in cell division (Willson et al., 2019). Paclitaxel resistance occurs *via* exosomal miR-30c by modulating EMT-linked molecules, including twinfilin 1 and interleukin-11 (Kutanzi et al., 2011). Inversely, exosomal miR-125b inhibits the semaphorin 4C (inducer of EMT) and enhances paclitaxel sensitivity. BCL2 and BCL2 homologous killer/antagonist [BAK1] types of apoptosis-linked molecules are also involved in the exosomal microRNA-mediated chemoresistance. Exosomal miR-125 modulates BAK1 and downregulates the paclitaxel-induced apoptosis, and exosomal miR-16/BCL2 enhances docetaxel sensitivity and results in increased apoptosis (Kastl et al., 2012).

### Hormonal agents

**FIGURE 2:**
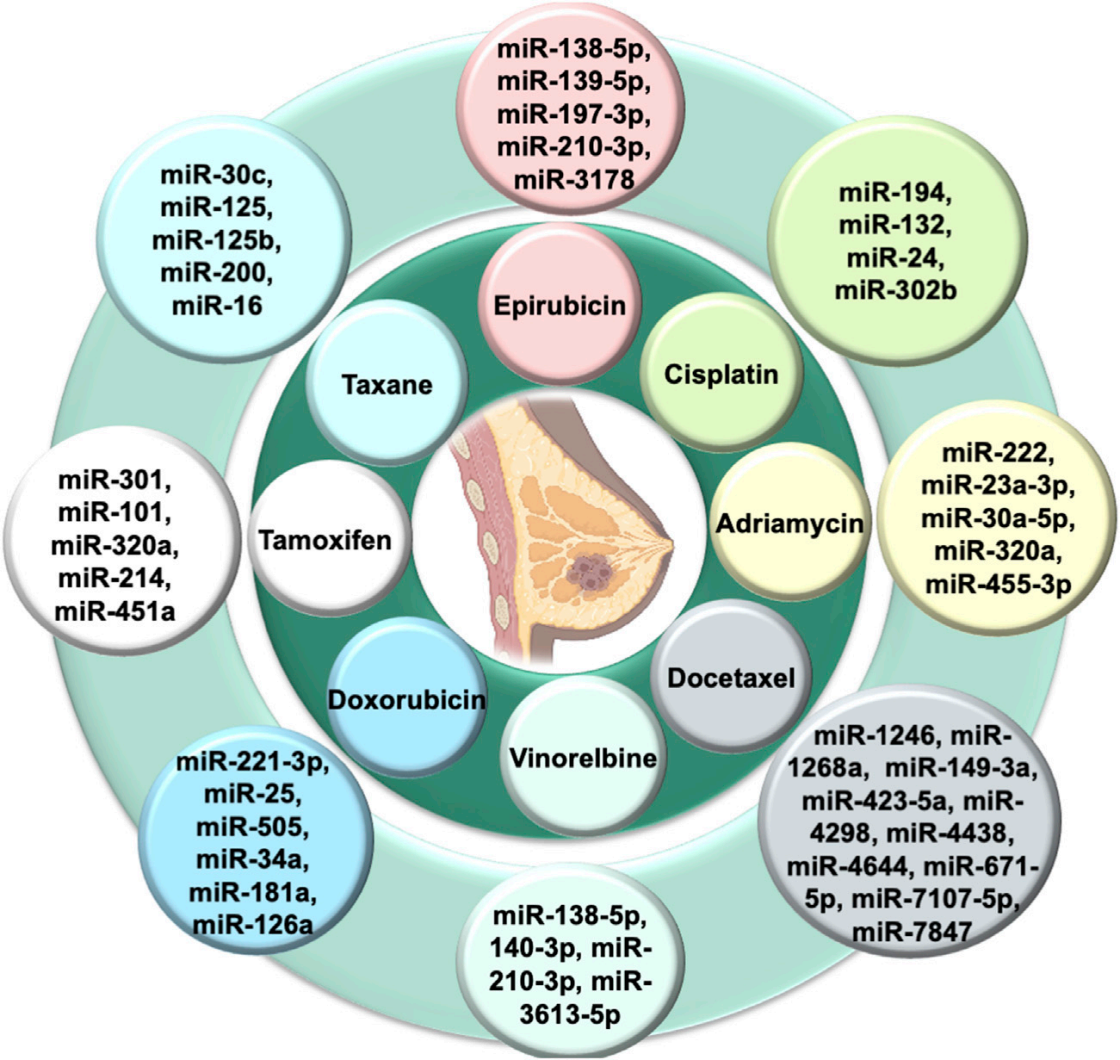
Exosomal miRNAs in chemoresistance of breast cancer.

Tamoxifen inhibits estrogen-mediated growth (Cui et al., 2015). PTEN regulates the PI3K/Akt/mTOR signal transduction pathway. Loss of the function of PTEN (inhibitor of PI3K) promotes chemoresistance (Nagata et al., 2004). Exosomal miR-101 suppresses the PTEN, resulting in the activation of Akt by regulating membrane-linked guanylate kinase [MAGI-2] to impart tamoxifen resistance in breast cancer (Sachdeva et al., 2011). Autophagy is also a crucial factor in maintaining homeostasis and is linked to chemoresistance. Exosomal miR-214 inhibits uncoupling protein 2 [UCP2]-dependent autophagy and restores the tamoxifen response to cancer cells (Yu et al., 2015)**.** Exosomal miR-221/222 promotes fulvestrant resistance in breast cancer cell lines by targeting β-catenin and TGF-β (Rao et al., 2011)**.** Exosomal miR-205-5p sensitizes breast cells to trastuzumab by modulating ERBB2 and targeting the P63/EGFR axis (De Cola et al., 2015) Figure 3) shows the role of various exosomal miRNAs in breast cancer chemoresistance (Figure 2).

## Exosomal miRNAs in BC invasion, migration, and metastasis

**FIGURE 3:**
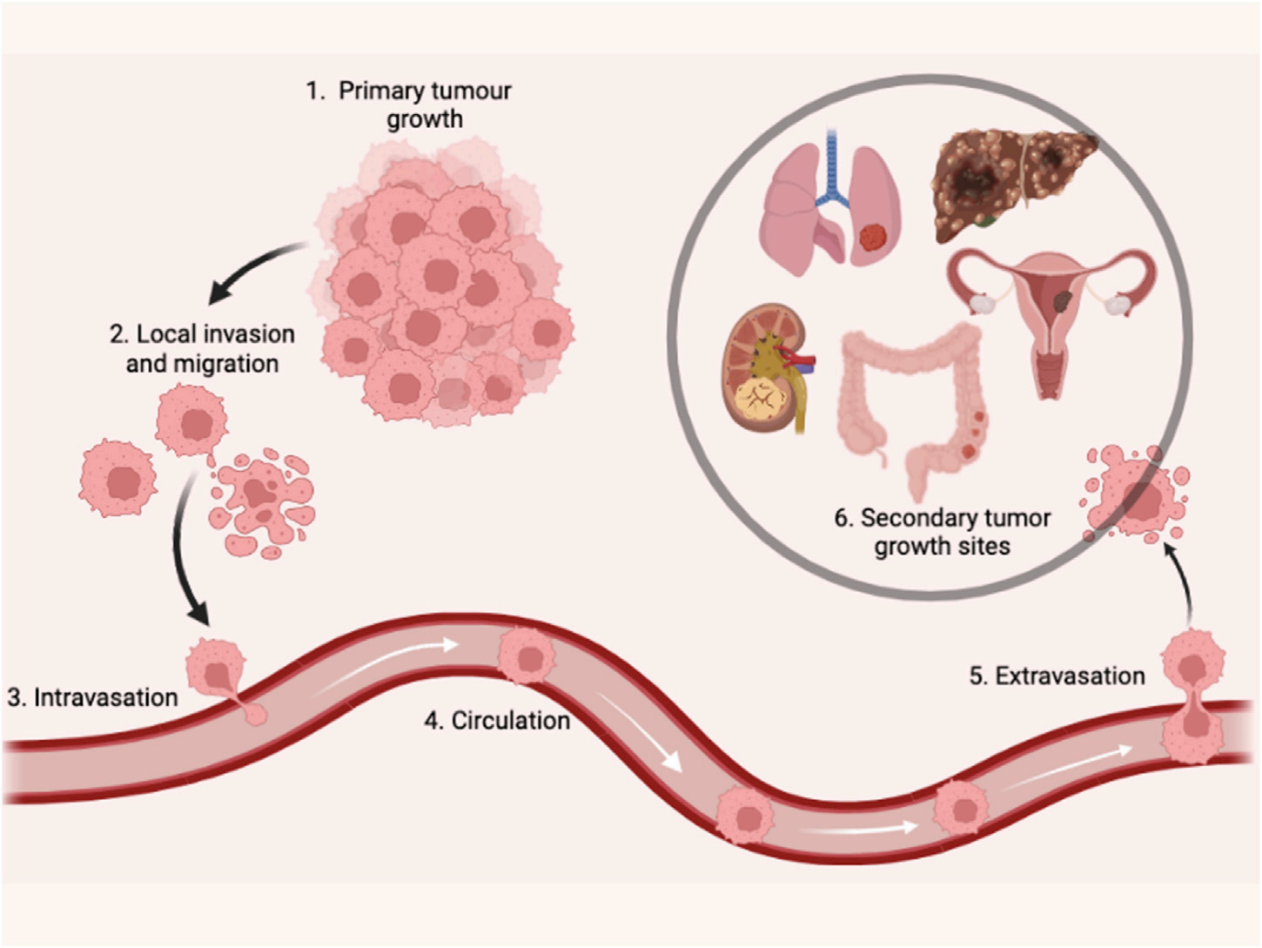
Illustrate the step of tumor growth into metastasis.

Metastasis is a pivotal factor for poor overall survival in breast cancer patients. It has been reported that exosomal miRNAs play a crucial role in almost every step of many biological processes in breast cancer (Liu et al., 2019; Figure 3). Many studies have demonstrated the dual role of exosomal miRNAs on breast cancer metastasis and related processes. In Table 2, we have mentioned some exosomal miRNAs along with their underlying mechanisms that are actively involved in metastasis, invasion, and migration. Table 2 shows the function of exosomal miRNAs in different hallmarks of BC, including invasion, migration, metastasis, stemness, and angiogenesis.

**TABLE 2:** Role of exosomal miRNAs in breast cancer invasion, migration, and metastasis.

Exosomal miRNA	Parameters	Molecular targets	Mechanism	References
miR-21, miR-10b, miR-373, miR-1246, miR-17-5p, miR-96, and miR-106b	Promotes invasion and migration	PTEN, PI3K, mapsin, PDCD4, HODX10, TBX5, DYRK1A, Syndecan-1, CCNG2, CD44, HBP1, TCF, LEF, ErbB2, FUT6, and Wnt/β-Catenin	┬ Apoptosis, ┬ clone formation, ↑ Tumorigenesis, ↑ Migration, and regulates cytoskeleton and E-cadherin	Huang et al. (2008), Zhu et al. (2008), Li et al. (2011), Ibrahim et al. (2012), Chen et al. (2013), Eichelser et al. (2014), Singh et al. (2014), Feng and Tsao (2016), Hong et al. (2016), Kim et al. (2016), Li et al., 2016; Shi (2016), Li et al. (2017b), and Xu et al. (2019)
miR-564, miR-10a, miR-34c, miR-217, miR-1226-3p, miR-21, miR-19a-3p, miR-148b-3p, miR-19b, miR-1486-3p, miR-148a, miR-503, miR-17/20, and miR-100	Suppresses invasion and migration	Akt, GNA12, GYS1, SRF, PIK/MAPK, mTOR, GIT1, KLF5, FZD8, Wnt-β-Catenin, AQP5, FOSL1, mucin1, TRIM29, CCND2/CCND3, E2F1, IL-8, and CCND1	Arrest cell cycle, ↑ apoptosis, ↑ Intracellular adhesion, and ┬ cell survival and growth	Yu et al. (2010), Bovy et al. (2015), Jiang et al. (2016), Mutlu et al. (2016), Tao et al. (2016), Ke and Lou (2017), Zhou et al. (2017), Yuan et al. (2019), and Park et al. (2020)
miR-10b, miR-503, miR-122, miR-200, miR-105, and miR-21	Promotes distant metastasis	β-Catenin, Twist, HOXD10, ROCK, c-Jun, XIST, STAT3, NF-ĸB, PD-L1, PKM, GLUT1, Sec23a, YAP1, ZO-1, LZTFL1, and EMT	┬ Growth, ┬ Local immunity, ↑ EMT, motility, modulates cytoskeletal flexibility, and ↑ cell proliferation	Ma, 2010; Ma et al. (2010), Korpal et al. (2011), Liu et al. (2012), Yu et al. (2013), Zhou et al. (2014), Fong et al. (2015), Knirsh et al. (2016), Xing et al. (2018), and Wang et al. (2019)
miR-193a, miR-124-3p, miR-720, miR-31, miR-429, miR-124, and miR-1	Suppresses distant metastasis	Wnt-β-Catenin, ZEB1, CRKL, CrKL, MMP-9, PDC D6, E-cadherin, Fzd3, Rho A, ITGA5, IL-11, Frizzled 7, TNKS2, BCL2, EGFR, WTL, TWISR1, HER2, Vimentin, and N-Cadherin	┬ EMT, ┬ cell motility, ↑ apoptosis, and impair tumorigenesis	Barrett-Lee (2009), Lv et al. (2011), Li et al. (2013), Li et al. (2014), Liu et al. (2015), Ye et al. (2015), Xie et al. (2017), Cai et al. (2018), Zhang et al. (2018), Peng et al. (2020), and Zhang et al. (2020)
miR-22, miR-221/222, miR-143, miR-21, and miR-378e	Promotes stemness	TET, PTEN, Akt, NF-ĸB, COX-2, Sox2, Oct3/4, nanog, Zeb, and Snail	↑ Stemness biomarkers, ↑EMT, and induces clonal expansion	Song et al. (2013), Li et al. (2017a), and Donnarumma et al. (2017)
miR-34a and miR-140	Suppresses stemness	NOTCH1, Sox2, Sox9	┬ Stem cells, modulate stem cell renewal, and shrink stem cells	Park et al. (2014), Wolfson et al. (2014), Kim et al. (2016)
miR-155 and miR-132	Promotes angiogenesis	VHL/HIF, RAS, and VEGF	↓ Pro-angiogenetic substrates	Kong et al. (2014) and Kontomanolis et al. (2017)
miR-16, miR-503, and miR-100	Suppress angiogenesis	VEGF, FGF2, VEGFA, mtor, and HIF-1α	Modulate expression of pro-angiogenic molecules and ┬ angiogenesis	Lee et al. (2013), Zhou et al. (2013), and Pakravan et al. (2017)

↑- Increase/upregulation; ↓- Decrease/Downregulation; ┴- Inhibit/Prevent.

## Exosomal microRNAs in the stemness of breast cancer cells

Cancer stem cells are groups of undifferentiated cells that have the potential to differentiate. Self-renewing populations of cells give rise to tumor heterogeneity, promoting metastasis, therapy resistance, and tumor recurrence (Vlashi and Pajonk, 2015). Breast cancer stem cells (BCSCs) mediate drug resistance and tumorigenesis because they are mostly found in the quiescent G0 phase, have high DNA repairability, and increase ABC transporter expression (Zhang et al., 2022). Exosomal miRNAs play a pivotal role in cancer stem cell maintenance. Exosomal miR-221/222 inhibits the PTEN activity that, in turn, activates the Akt/NF-κB/COX-2 signal transduction pathway and promotes stemness-like traits in breast cancer cells (Li B. et al., 2017). Exosomal miR-22 suppresses the TET/miR-200 axis *via* inhibiting TET [ten eleven translocation-DNA demethylase family], resulting in increased stemness and EMT (Song et al., 2013). Exosomal miRNAs target stemness by modulating the function and expression of breast cancer stemness-linked genes and their elements. Exosomal miR-34a downregulates the NOTCH1 expression in breast cancer, leading to decreased stemness in the mammosphere (Park et al., 2014). Sox9 is an oncogenic transcription factor that induces the transformation of mammary stem cells from differentiated mammary epithelial cells, which is crucial for breast cancer initiation and malignancy (Guo et al., 2012). Exosomal miR-140 decreases the expression of SOX2/SOX9 and reduces the stem cell population and disordered stem cell renewal (Wolfson et al., 2014).

## Exosomal microRNAs in angiogenesis of breast cancer cells

**FIGURE 4:**
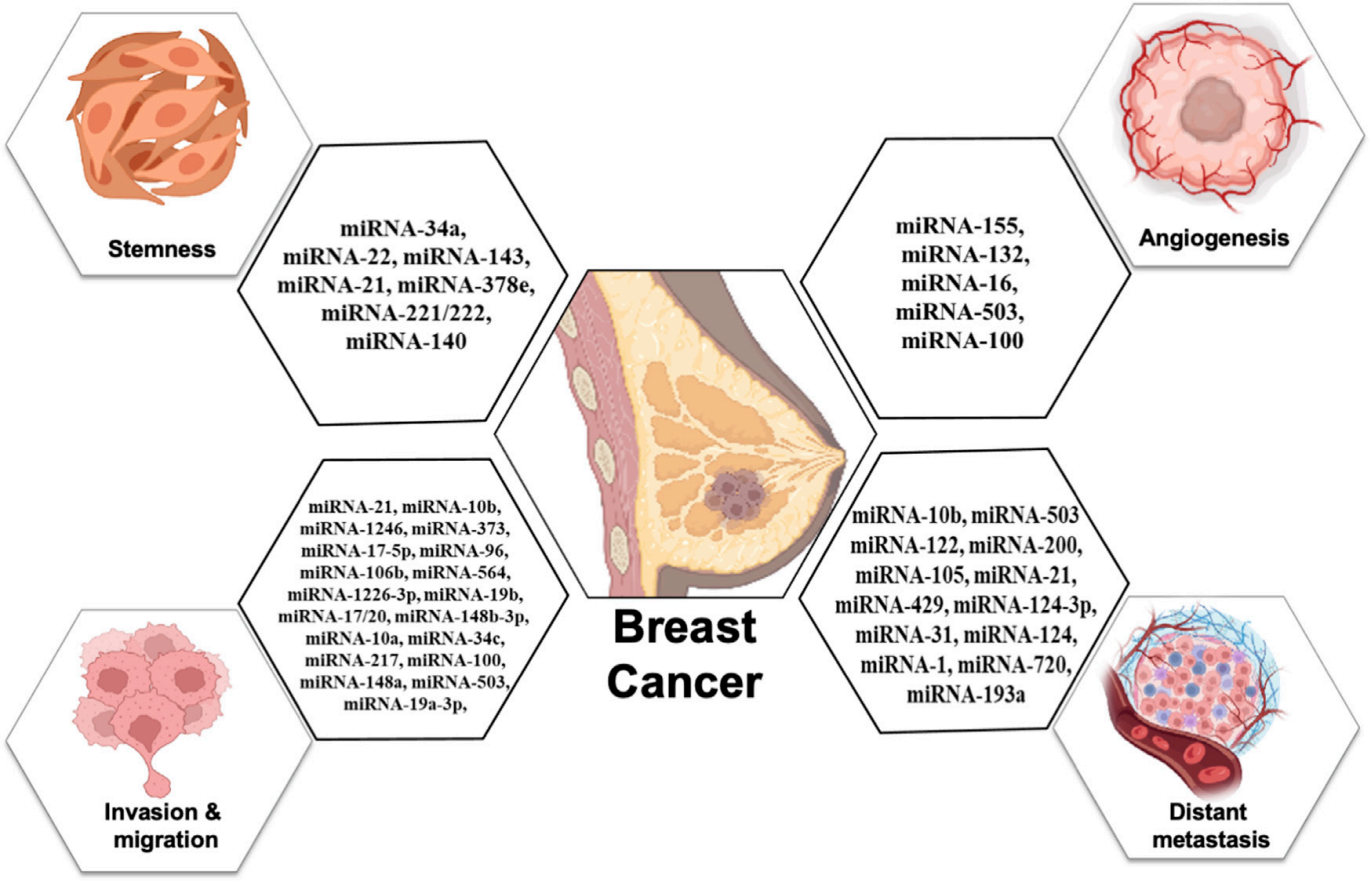
Role of exosomal miRNAs in different hallmarks of breast cancer.

Exosomal miRNAs play a significant role in angiogenesis to reduce the metastasis of breast cancer. Kong et al. (2014) reported that exosomal miR-155 modulates the Von Hippel–Lindau [VHL]/hypoxia-inducible factor and its downstream genes, including pyruvate kinase isozyme type M2, CD44, interleukin 6, and vascular endothelial growth factor in an *in vivo* mice model (Kong et al., 2014). Exosomal miR-132 enhances the sensitivity of endothelial cells to VEGF by decreasing the activity of the RAS suppressor, augmenting angiogenesis (Kontomanolis et al., 2017). Exosomal miR-16 halts the expression of VEGF by playing an anti-angiogenic role (Lee et al., 2013). Some exosomal miRNAs also target the tumor microenvironment and halt angiogenesis; for example, exosomal miR-503 regulates the expression of VEGFA and fibroblast growth factor 2 and hampers angiogenesis (Zhou et al., 2013)**.** Exosomal miR-100 decreases the expression of human umbilical vein endothelial cells [HUVECs] and targets mTOR/[HIF-1α]/VEGF to decrease angiogenesis (Pakravan et al., 2017). Figure 4 shows the role of different exosomal miRNAs in different hallmarks (invasion and migration, distant metastasis, stemness, and angiogenesis) of breast cancer.

## Conclusion and future perspective

Exosomal miRNAs play a significant role in almost all hallmarks of breast cancer, including chemoresistance, metastasis migration, invasion, stemness, and angiogenesis. Exosomal miRNAs show a dual mode of action w.r.t to chemoresistance and drug sensitivity. As breast cancer has many subtypes with different prognostic and clinicopathological features, it is necessary to examine the specific miRNA for multiple subtypes to evaluate the targeted therapeutic regime in breast cancer. The exosomal content w.r.t. miRNA increases to varying degrees that distinguish healthy controls from breast cancer patients and TNBC from other subtypes. Exosomal miRNAs may serve as an indication of the early stage of breast cancer; for example, exosomal miR-221/222 is found in almost every stage of breast cancer oncogenesis, and this exosomal miRNA-221 can predict the breast cancer origin, progress, and treatment effect. In stages I and II of breast cancer, miR-801, miR-127-3p, miR-148b, and miR-409-3p increased significantly, which is helpful for early detection.

The equilibrium types and numbers of exosomal miRNAs vary in pathological conditions; they serve as useful biomarkers for breast cancer prognosis and diagnosis. Exosomal miRNAs exert pleiotropic impacts on breast cancer hallmarks and clinical implications. (Luo et al. (2023) revealed the role of tumor-derived exosomes as a messenger and communicator between tumor cells and their microenvironment that can reshape and enhance tumor development and metastasis. They enhance the development of the pre-metastatic niche to promote tumor colonization and play a pivotal role in the early diagnosis and evaluation of future metastatic development (Luo et al., 2023). Exosomes are used as drug carriers (e.g., chemotherapy and miRNA) and as biomarkers because they exhibit antigen-presenting traits that have a crucial function in cancer immunotherapy (Hosseinikhah et al., 2023). *Prakriti* [phenotype-associated Ayurveda constitution] is linked with other fields like genomics, physiology, psychology, and therapeutics. It has a role in the evaluation of therapeutics such as early diagnosis, indicating disease susceptibility, prevention of diseases, drug design, and customization of therapy [lifestyle, drug, and diet]. This holds great potential for personalized medicine pharmacogenomics for predictive or preventive medicine (Sharma and Prajapati, 2020). This review gives a new insight that helps us examine prognostic, predictive, and diagnostic markers at the cellular and molecular levels to fill research gaps for novel therapeutic approaches and for future personalized medicine.
